# *Brucella* Genital Tropism: What's on the Menu

**DOI:** 10.3389/fmicb.2017.00506

**Published:** 2017-03-28

**Authors:** Jean-Jacques Letesson, Thibault Barbier, Amaia Zúñiga-Ripa, Jacques Godfroid, Xavier De Bolle, Ignacio Moriyón

**Affiliations:** ^1^Research Unit in Microorganisms Biology, University of NamurBruxelles, Belgium; ^2^Facultad de Medicina, Departamento de Microbiología y Parasitología, Edificio de Investigación, Instituto de Salud Tropical e Instituto de Investigación Sanitaria de Navarra, Universidad de NavarraPamplona, Spain; ^3^Arctic Infection Biology, UiT - The Arctic University of NorwayTromsø, Norway

**Keywords:** *brucellosis*, tropism, placenta, epididymis, metabolism, erythritol, lactate, glutamate

If things such as Tripadvisor web-site or Foursquare apps existed for bacteria, for sure, to the question “*What is the best place to eat near me*?” or “*Where can I find my favorite food?*” *Brucella* would be advised “*male and female genital organs*” as a first choice with millions of positive comments from previous and highly satisfied congeneric visitors. The friendly ambiance and the relish for the specialties of the chef are illustrated by the fact that the infected bovine concepts can host up to 10^14^ brucellae (Alexander et al., 1981; Corner, 1983).

As expected, these bacteria are not welcome visitors: they cause brucellosis, a highly contagious zoonosis that affects many species of wild and domestic animals (Zheludkov and Tsirelson, 2010; Atluri et al., 2011) and represents a serious burden for livestock and humans worldwide (McDermott et al., 2013). In their hosts, these gram-negative coccobacilli replicate in an endoplasmic reticulum-derived vacuole of professional (macrophages, dendritic cells) and non-professional (e.g., trophoblastic cells) phagocytes (Atluri et al., 2011), and in sexually mature animals have a pronounced tropism for genital organs causing orchitis, epididymitis and infertility in males, and abortion (most often in the last third of gestation) and sterility in females (Moreno and Moriyón, 2006). This genital tropism also holds true in humans as *Brucella* induces epididymo-orchitis and can infect the placenta even if abortion is rare (Anderson et al., 1986; Queipo-Ortuño et al., 2006). Sexual secretions and aborted tissues of animals contain billions of bacteria and, as the brucellae cannot survive long in the environment, these high numbers grant transmission via aerosols, ingestion or sexual intercourse (Poester et al., 2013). Therefore, the localization and abundant multiplication in the reproductive tract of animals is crucial in the biology of this parasite.

Although identified as a characteristic of the disease in ruminants by the end of the Nineteenth century (reviewed in Huddleson et al., 1943), the reason(s) underlying the genital tropism of *Brucella* and subsequent intense multiplication are still to be deciphered. Clearly, any unifying hypothesis should consider properties shared by both the male and female organs of the target species, which rules out some possibilities. For instance, although *Brucella* is carried by blood cells (Anderson et al., 1986; Vitry et al., 2014), the (erythro-)phagocytic properties of the trophoblasts as an entry gate to the placenta cannot be invoked to explain the localization in seminal vesicles. On the other hand, immune privilege (Filippini et al., 2001) in testis and semen or local immunosuppression at the feto-maternal interface (Warning et al., 2011) could well be part of the explanation. Yet, tolerance cannot be enough: an intense multiplication needs timely and effective sources of carbon, nitrogen and energy. Following this idea, we will argue here that some peculiarities of the metabolism of these organs provide nutrients that match the metabolic abilities of *Brucella* and are thus playing a prominent role.

Although, the link with *Brucella* metabolism may not be immediately evident, female and male genital organs have some common metabolic features, the most obvious one being a rather high production of fructose. This is, for example, the case of epitheliochorial and synepitheliochorial placentas (e.g., those of cows, ewes and sows) where fructose is in fact the major sugar. To a lesser extent, fructose is also present in other types of placentas like those of dogs, cats, guinea pigs, rabbits, rats, and ferrets (Alexander et al., 1955; Håstein and Velle, 1968; Battaglia and Meschia, 1978; Reitzer et al., 1979; Kim et al., 2012). A similar dominance of fructose over glucose occurs in epididymes, seminal fluids and oviducts of several mammals (boars, bulls, rats, guinea pigs, etc.) where it serves as the primary energy source for spermatozoids (Frenette et al., 2004; Frenette, 2006; Pruneda et al., 2006; Larose et al., 2012). In all these organs, fructose originates from glucose through the Polyol Pathway, initially described in the fetal tissues of ungulates (Hers, 1956). In this pathway, glucose is reduced to sorbitol in a reaction catalyzed by the aldose reductase (AR) and this C6 polyol is then oxidized to fructose by sorbitol dehydrogenase, which uses NAD as electron acceptor. Since AR uses as proton/electron donor the NADPH furnished by the Pentose Phosphate (PP) Pathway (or hexose monophosphate shunt), there is a connection between the PP and Polyol pathways. Fructose, however, does not support growth of all *Brucella* strains (McCullough and Beal, 1951), and what are noteworthy here are some intricacies of these two pathways.

The PP pathway is known to occur, among other places, in the testes, ovaries, adrenal cortex, and placenta where the NADPH it produces is crucially needed for the synthesis of steroid hormones (Ferrier, 2013). In 1967, Clark et al. (1967) proposed that this pathway was involved in the production of erythritol, a C4 sugar alcohol they identified among other polyols in bovine semen. This suggests a prominent albeit indirect role for the PP pathway in *Brucella* genital tropism because previous work not only had shown that erythritol is the preferred carbon/energy source of *B. abortus, B. melitensis* and *B. suis* (McCullough and Beal, 1951) but also had reported that it displayed “vitamin-like” properties stimulating the growth of these *Brucella* spp. in catalytic amounts (Keppie et al., 1965). Since then, the high concentrations of erythritol in fetal fluids, placental tissue, epididymis and semen of the preferred hosts of those *Brucella* species have been postulated as important in the genital tropism of these pathogens in ruminants (Smith et al., 1962; Clark et al., 1967; Essenberg et al., 2002) and, indeed, recent evidence strongly suggests it is presence in various concentrations in cells and tissues of other *Brucella* hosts where it was not detected previously (Lowrie and Kennedy, 2001; Burkhardt et al., 2005; Jauniaux et al, 2005). Moreover, speculating on the origin of erythritol in bovine fetal fluids, where it was first identified in animal tissues, Pearce had the insight that: “*it may arise from D-erythrose, a possible product of the pentose phosphate pathway, and act as an intermediate between D-erythrose and D-erythrulose as sorbitol acts as an intermediate between glucose and fructose*” (Pearce et al., 1962). In fact, in bovine genital organs erythritol concentration parallels that of fructose and is very likely produced by the action of AR because, in addition to a very broad ability to reduce aldehydes to their corresponding alcohols, its Km is far lower for D-erythrose (4 10^−4^ M/L) than for glucose (7 10^−2^ M/L) (Hayman and Kinoshita, 1965). AR would thus bridge the Polyol and PP pathways by both acting on a PP sugar and using a PP coenzyme.

It is worth noting that AR activity increases during pregnancy (Håstein and Velle, 1968). Thus, erythritol production could rise following a dynamics that overlaps with the increased susceptibility to *Brucella* colonization that occurs during the second half of pregnancy, as shown by field experience, repeated observations with *B. melitensis* vaccine Rev 1 (which displays a comparatively high residual virulence, Blasco, 1997) and controlled experiments in cattle (Crawford et al., 1987). Studying chorioallantoic membrane explants, Samartino and Enright found that the replicative capability of the bacteria was better in late gestational placental tissue than in tissue harvested earlier (Samartino and Enright, 1992). Later on they found that erythritol production was highest at mid pregnancy (Samartino et al., 1994). However, they pointed out also that erythritol was unlikely to be the only or even main factor operating in their *in vitro* system as experiments with *B. abortus* S19, which is inhibited by erythritol, yielded results not different from those obtained with the virulent strain (Samartino and Enright, 1992). Even though an interpretation is obscured by the isolation of erythritol-resistant mutants of S19 (Corner and Alton, 1981), this vaccine is still able to induce abortions in pregnant cattle (albeit only in a low proportion; Nicoletti, 1976), which also suggests the existence of other metabolic factors that would allow its efficient growth in placentas.

In this context, it is important to note that, although erythritol is a *Brucella* preferential carbon source, the placenta and the male genital organs and fluids also have high concentrations of glycerol, lactate, and glutamate (Figure 1).

**Figure 1:**
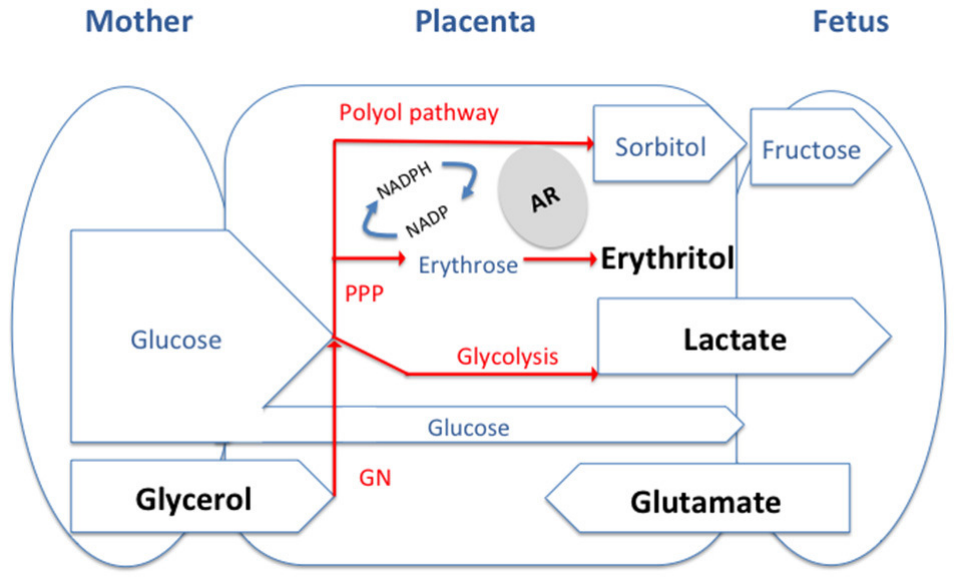
**General model of the feto-maternal interface and of the main carbon source available for *Brucella*.** The substrates we discuss in the paper are in Bold and are likely to be present at different levels in tissues of different host depending on the type of placentation. PPP, pentose phosphate pathway; GN, gluconeogenesis; AR, aldose reductase.

In late pregnancy, due to the enhanced adipose tissue lipolytic activity, the maternal plasma concentration of glycerol is consistently elevated. Because of the rapid and efficient conversion of maternal glycerol into glucose there is little direct glycerol transfer to the fetus by the placenta (Palacin et al., 1987). The glycerol gradient between maternal plasma and fetal blood is greater in species with epitheliochorial placentation (i.e., ruminants) than in accidental hosts that like humans have a hemochorial placenta (Herrera, 2002). Glycerol has also been described, either free or as glyceryl-phosphoryl-choline, in bull and ram semen where it is taken by spermatozoa and metabolized through an oxidative process into lactate (Britton, 1962; Clark et al., 1967). Concerning the latter substrate, the placenta of different mammals, even under aerobic conditions, produces lactate in relatively large amounts, presumably from maternal glucose, and supplies it to the fetus where it is an important metabolic substrate. Likewise, male germ cells preferentially use lactate and pyruvate over glucose as an energy substrate (Battaglia and Meschia, 1978; Père, 2003; Goldberg et al., 2010). Finally, glutamate is also available in genital tissues of at least sheep. While it appears that neutral and basic amino acids are transported from the ovine placenta into fetal blood, the acidic amino acids glutamate and aspartate are not. In fact, glutamate is delivered by the fetal lamb to the placenta in large amounts and then converted to glutamine before being released back into the fetal circulation (Battaglia and Meschia, 1978; Wu et al., 2015). Glutamate is also the main free amino acid in the testis and semen of most mammalian species, where it occurs mainly in the seminal plasma (Setchell et al., 1967; Keil et al., 1979).

Amazingly glutamate, lactate and glycerol match the three carbon (and nitrogen for glutamate) sources identified by Gerhardt et al. (1950) as suitable to formulate a defined minimal medium for *Brucella* growth that obviated the need for complex amino acid mixtures. Gerhardt et al. reported that only glutamate among 20 amino acids and related nitrogen compounds was used as sole source of carbon, nitrogen and energy by S19 and two wild-type *B. abortus* strains (Gerhardt et al., 1950). Even though this is not true for all *B. suis* strains and at least some *B. abortus* and *B. melitensis* strains are respectively auxotrophic for specific amino acids or require a CO_2_ supply for growing on glutamate (Plommet, 1991), the fact stands that all *Brucella* species have in common a remarkable capacity to oxidize exogenous glutamate (Verger and Grayon, 1977; Jacques et al., 2007). As expected, genomic and mutant analysis show that the brucellae keep a conventional enzymatic machinery for using glutamate both for amino acid synthesis and as a C source feeding the Krebs cycle (Ronneau et al., 2014).

Although, reports on lactate as a source of C for *Brucella in vitro* are few, it has been noted that it is very effectively used and that it can even advantageously replace glucose (Gerhardt and Wilson, 1948). These effects can be explained, at least in part, by the existence of a lactate permease and a primary L-lactate dehydrogenase coupled to the electron transport chain whose efficiency is similar to that of the primary erythritol-1-phosphate dehydrogenase (Rest, 1975). Similarly, there are few studies on glycerol *in vitro*, and it is unclear whether the growth promoting effect observed by Gerhard and Wilson relate only to an ability to act as an excellent complementary C source for lactate (Gerhardt and Wilson, 1948). On one hand, the rather high concentration of glycerol in Gerhard and Wilson's medium seems metabolically unnecessary and it has been interpreted to mean that non-nutritional properties, such as a proper control of the E_h_ of the medium, could contribute (Gerhardt, 1958). On the other, even though impurities such as vitamins or amino acids in the reagent used in early studies cannot be ruled out (Gerhardt, 1958; Plommet, 1991), recent observations *in vitro* also support the complementarity role of glycerol (Zúñiga-Ripa et al., 2014).

It seems, therefore, a plausible hypothesis that there is an adequacy between the presence and abundance of privileged nutrients in the male and female genitals organs and the nutritional preferences of *Brucella* in some simple defined media. Strong candidates are erythritol, lactate, glycerol and glutamate, in all likelihood in various combinations and proportions. To test this hypothesis would require assaying defined metabolic mutants for their residual virulence in reproductive tissues (e.g., male or female genital tissue colonization, induction of fetal pathology and abortion) of natural hosts. Regrettably, in most countries, governmental regulations represent a bottleneck for this kind of research that is almost impossible to circumvent and, although imperfect, the mouse model remains as the only feasible surrogate.

It has been suggested that rather than facultative intracellular parasites, the brucellae should be described as intracellular facultative extracellular bacteria (Moreno and Moriyón, 2002) and, indeed, their ability to persist outside the host is very limited at least for the classical species. As emphasized above, they circumvent their precarious condition in the environment by being released in numbers so high that make contagion highly probable. From this perspective, the coincidences discussed here are unlikely to be accidental. If our hypothesis is proven true, the identification of erythritol, lactate, glycerol and glutamate as effective substrates for *Brucella* growth *in vitro* would be more than a mere coincidence as it would reflect metabolic preferences suitable for a pathogen that relies on genital tropism to close its biological cycle. These bacteria have been described as “stealthy” because of their ability not to be detected effectively by innate immunity. Could we also consider them as “gourmet” or “greedy” to describe a key step of their behavior in their hosts?

## Author contributions

JJL, TB, IM, AZR, XDB, and JG had discussion about the content and corrected the paper. JJL and IM wrote the paper.

### Conflict of interest statement

The authors declare that the research was conducted in the absence of any commercial or financial relationships that could be construed as a potential conflict of interest.
